# Enhanced ventral hippocampal synaptic transmission and impaired synaptic plasticity in a rodent model of alcohol addiction vulnerability

**DOI:** 10.1038/s41598-017-12531-z

**Published:** 2017-09-26

**Authors:** Antoine G. Almonte, Sarah E. Ewin, Madelyn I. Mauterer, James W. Morgan, Eugenia S. Carter, Jeffrey L. Weiner

**Affiliations:** Department of Physiology and Pharmacology, Wake Forest School of Medicine, Medical Center Boulevard, Winston-Salem, NC 27157 USA

## Abstract

It has long been appreciated that adolescence represents a uniquely vulnerable period when chronic exposure to stressors can precipitate the onset of a broad spectrum of psychiatric disorders and addiction in adulthood. However, the neurobiological substrates and the full repertoire of adaptations within these substrates making adolescence a particularly susceptible developmental stage are not well understood. Prior work has demonstrated that a rodent model of adolescent social isolation (aSI) produces robust and persistent increases in phenotypes relevant to anxiety/stressor disorders and alcohol addiction, including anxiogenesis, deficits in fear extinction, and increased ethanol consumption. Here, we used extracellular field recordings in hippocampal slices to investigate adaptations in synaptic function and synaptic plasticity arising from aSI. We demonstrate that this early life stressor leads to enhanced excitatory synaptic transmission and decreased levels of long-term potentiation at hippocampal Schaffer collateral-CA1 synapses. Further, these changes were largely confined to the ventral hippocampus. As the ventral hippocampus is integral to neurocircuitry that mediates emotional behaviors, our results add to mounting evidence that aSI has profound effects on brain areas that regulate affective states. These studies also lend additional support to our recent proposal of the aSI model as a valid model of alcohol addiction vulnerability.

## Introduction

A large and compelling body of research in human populations and animal models supports the idea that exposure to chronic early-life stress (e.g., social isolation during adolescence) can catalyze the development of psychiatric disorders and addiction in adulthood^1–4^. Thus, it is widely accepted that adolescence is a critical and particularly vulnerable stage of neurological development during which exposure to stressors can cause persistent changes in brain function and behavior. To begin to better understand the neurocircuitry and neurobiology underlying this unique vulnerability and the transition to alcohol addiction, our lab has endeavored to characterize a simple rodent adolescent social isolation (aSI) model that engenders robust and enduring increases in many behavioral risk factors of alcohol addiction, including increased anxiety-like behaviors and ethanol intake^5^.

Intense investigation by many groups suggests that the basolateral amygdala (BLA) and the hippocampus (HC) are critical components of a distributed network that subserves the processing of stimuli and the gating of behavioral responses underlying stress, fear, and anxiety-like behaviors^6–9^. Modulating the balance between excitatory to inhibitory activity within either of these brain regions can drive behavioral output and induce synaptic plasticity. For example, our lab and others have previously demonstrated that increasing BLA pyramidal cell activity is correlated with the expression of anxiety-like behaviors, while decreasing activity of these cells is associated with reductions of these behaviors^4,9–12^. Further, much accumulated evidence demonstrates that exposure to acute and chronic stress results in aberrant excitatory activity, which in turn, impairs the induction of long-term plasticity between the BLAandHC, and also impairs hippocampus-dependent spatial learning, and extinction of learned fear^13–17^. Enhanced excitatory activity has also been observed with chronic exposure to ethanol^18–20^. Indeed, observations of excitatory/inhibitory imbalances have become a recurring theme in the stress, fear, anxiety, and alcohol addiction literatures, lending further support to the idea that these conditions may share common underlying mechanisms^21^.

Although the hippocampus has been commonly viewed as a singular structure subserving cognition and memory, a growing body of literature supports the concept of the hippocampal formation as a set of functionally segregated domains, with the dorsal hippocampus (dHC) participating in the encoding of declarative memories, and the ventral hippocampus (vHC) intimately involved in the gating of emotional behaviors and stress responses^7,22–24^. Anatomical studies have described a strong projection from the BLA to the vHC, which suggests an important functional relationship between these two brain areas^25–30^. Indeed, burgeoning evidence implicates BLA-vHC connectivity as critical for mediating contextual associations in fear memory formation and extinction, and in modulating the expression of anxiety-related and social behaviors^10,31–39^. Our lab has also recently reported that adolescent social isolation leads to a significant increase in the intrinsic excitability of BLA pyramidal neurons^12^. However, little is known about the effects of adolescent social isolation in brain regions, like the hippocampus, that receive strong BLA innervation and no studies to date have specifically examined whether adolescent social isolation exerts differential effects on dorsal and ventral hippocampal subregions.

Here, we conducted an examination of the effect of adolescent social isolation on hippocampal synaptic transmission and synaptic plasticity. Specifically, we tested the hypothesis that adolescent social isolation would lead to increases in measures of excitatory synaptic transmission and impaired long-term synaptic plasticity. This examination revealed that adolescent social isolation resulted in an increase in baseline synaptic transmission and a decrease in theta-burst stimulation (TBS) induced long-term potentiation (LTP) in extracellular field recordings at Schaffer collateral-CA1 synapses. Based on the known differences between the dorsal and ventral domains of the hippocampus, we further examined the effects of adolescent social isolation within each subregion. This deeper analysis revealed an enhancement of synaptic transmission and a reduction of LTP levels that were largely restricted to the vHC, the hippocampal subregion that receives extensive BLA innervation. Together, these studies reveal that adolescent social isolation has differential effects on hippocampal subregions, primarily enhancing excitability and subsequently leading to an occlusion of LTP within the vHC. These initial finding suggest that adaptive changes within this “emotional” region of the hippocampus may contribute to the “addiction-vulnerable” phenotype that manifests following exposure to this early-life stressor.

## Results

### Adolescent social isolation enhances baseline synaptic transmission at hippocampal Schaffer collateral-CA1 synapses

**Figure 1:**
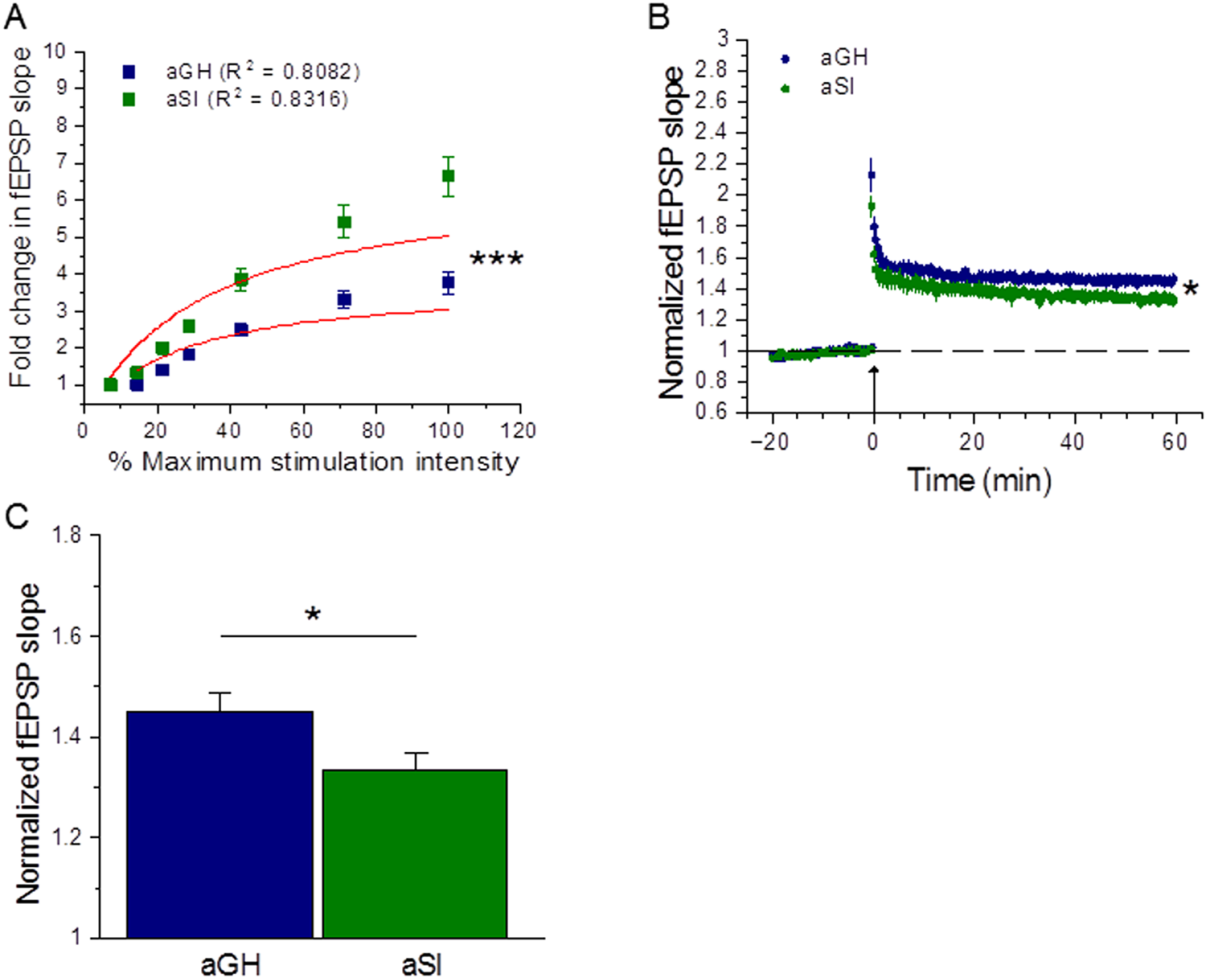
Adolescent social isolation enhances baseline synaptic transmission and reduces LTP at hippocampal Schaffer collateral-CA1 synapses. (**A**) aSI rats show significant increases in the input/output relationship (aGH: n = 60 slices, 29 rats; aSI: n = 44 slices, 28 rats; red lines indicate fit to Michaelis-Menten model, F_(1, 11)_ = 26.50, p < 0.001), F-test). (**B**) aSI rats display significantly reduced LTP magnitudes. Arrow indicates delivery of TBS (aGH: n = 50 slices, 28 rats; aSI: n = 34 slices, 19 rats). (**C**) Comparison of normalized fEPSP slopes averaged over the last ten minutes of recording reveal lower LTP magnitudes in aSI rats than aGH rats (p = 0.026, two-way RM ANOVA).

We have begun investigating the effects of adolescent social isolation on hippocampal synaptic function using extracellular field recordings in acute brain slices prepared from adolescent group housed ﻿(aGH) and adolescent socially isolated﻿ (aSI) rats. We first assessed baseline synaptic transmission by measuring field excitatory postsynaptic potential (fEPSP) magnitudes in response to increasing stimulus intensity levels to generate input/output curves. These experiments revealed an enhancement of synaptic responses in aSI rats compared to aGH rats, as plotted by fold change in fEPSP slope over the threshold stimulation intensity for eliciting a synaptic response (Fig. 1A; aGH: n = 60 slices, 29 rats; aSI: n = 44 slices, 28 rats). The input/output plots depict a linear rising phase that reaches a plateau, which are reminiscent of enzyme kinetic saturation curves; thus, these data were fit to a Michaelis-Menten model. As the red lines in Fig. 1A illustrate, a Michaelis-Menten model is a very good fit to each dataset (aGH: R^2^ = 0.8082, aSI: R^2^ = 0.8316). An F-test to compare curve fits revealed that input/output curves were significantly different between aGH and aSI rats (F_(1,11)_ = 26.50, p < 0.001, F-test). These results suggest that adolescent social isolation enhances baseline synaptic transmission at hippocampal Schaffer collateral-CA1 synapses.

### Adolescent social isolation reduces hippocampal long-term potentiation

As various models of stress have been demonstrated to have deleterious effects on hippocampal synaptic plasticity^13,15,17^, we assessed the effect of adolescent social isolation on the induction of long-term plasticity (LTP). Theta-burst stimulation (TBS) was chosen as the LTP induction protocol for several reasons. First, because TBS mimics the complex-spike discharges and modulation of pyramidal cell excitability obtained using *in vivo* recordings from awake animals during certain learning tasks, it is considered a physiologically relevant stimulus protocol^40–42^. Second, a number of studies have underscored the importance of BLA-HC communication via hippocampal theta rhythm in the encoding of memories and the gating of behavioral responses^43–45^. Finally, mechanisms underlying TBS-induced LTP at hippocampal Schaffer collateral-CA1 synapses have been extensively studied^41,46,47^, thereby providing well-characterized parameters for evaluating the effects of adolescent social isolation on LTP.

Our LTP studies demonstrate that adolescent social isolation led to a reduction in TBS-induced LTP levels (Fig. 1B; aGH: 1.45 ± 0.034, n = 50 slices, 28 rats; aSI: 1.33 ± 0.041, n = 34 slices, 19 rats). Comparing the averages of normalized fEPSP slopes for the last ten minutes of recording further emphasizes the lower LTP magnitudes in aSI rats (Fig. 1C, effect of housing (aGH vs. aSI): F_(1,80)_ = 5.17, p = 0.026, two-way repeated-measures ANOVA (two-way RM ANOVA)). Together with the input/output data, these results suggest that adolescent social isolation appears to enhance hippocampal baseline synaptic transmission and interferes with processes underlying the induction of long-term synaptic plasticity.

### Adolescent social isolation differentially affects baseline synaptic transmission, but does not affect short-term plasticity in dorsal and ventral hippocampus

**Figure 2:**
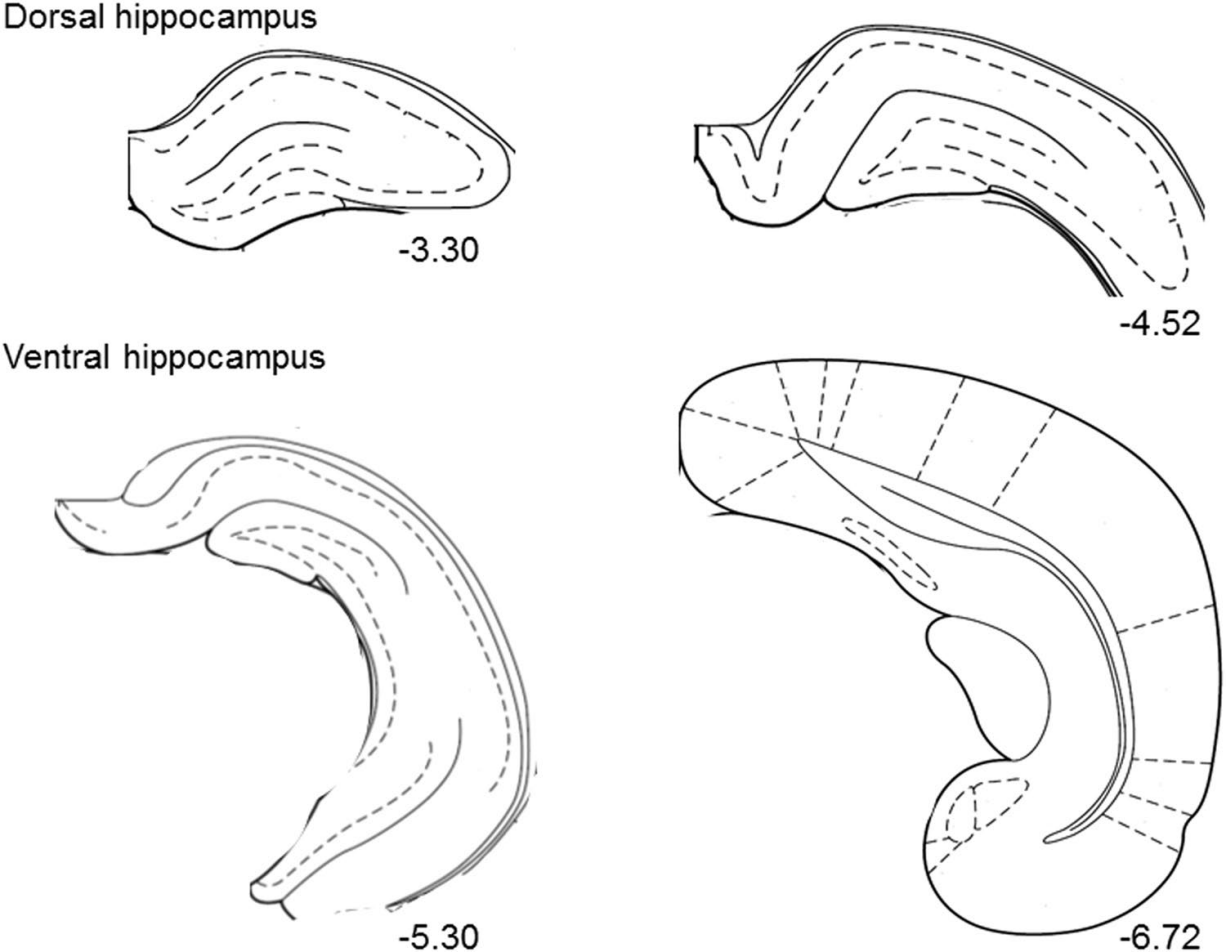
Examples of slices taken from dorsal and ventral hippocampus. Representative drawings depict slices taken from dorsal hippocampus (dHC) (top; range: Bregma −3.30 to −4.52 mm) and ventral hippocampus (vHC) (bottom; range: Bregma −5.30 to −6.72 mm) for extracellular field recordings. Numbers below each slice indicate coordinates from Bregma in mm.

**Figure 3:**
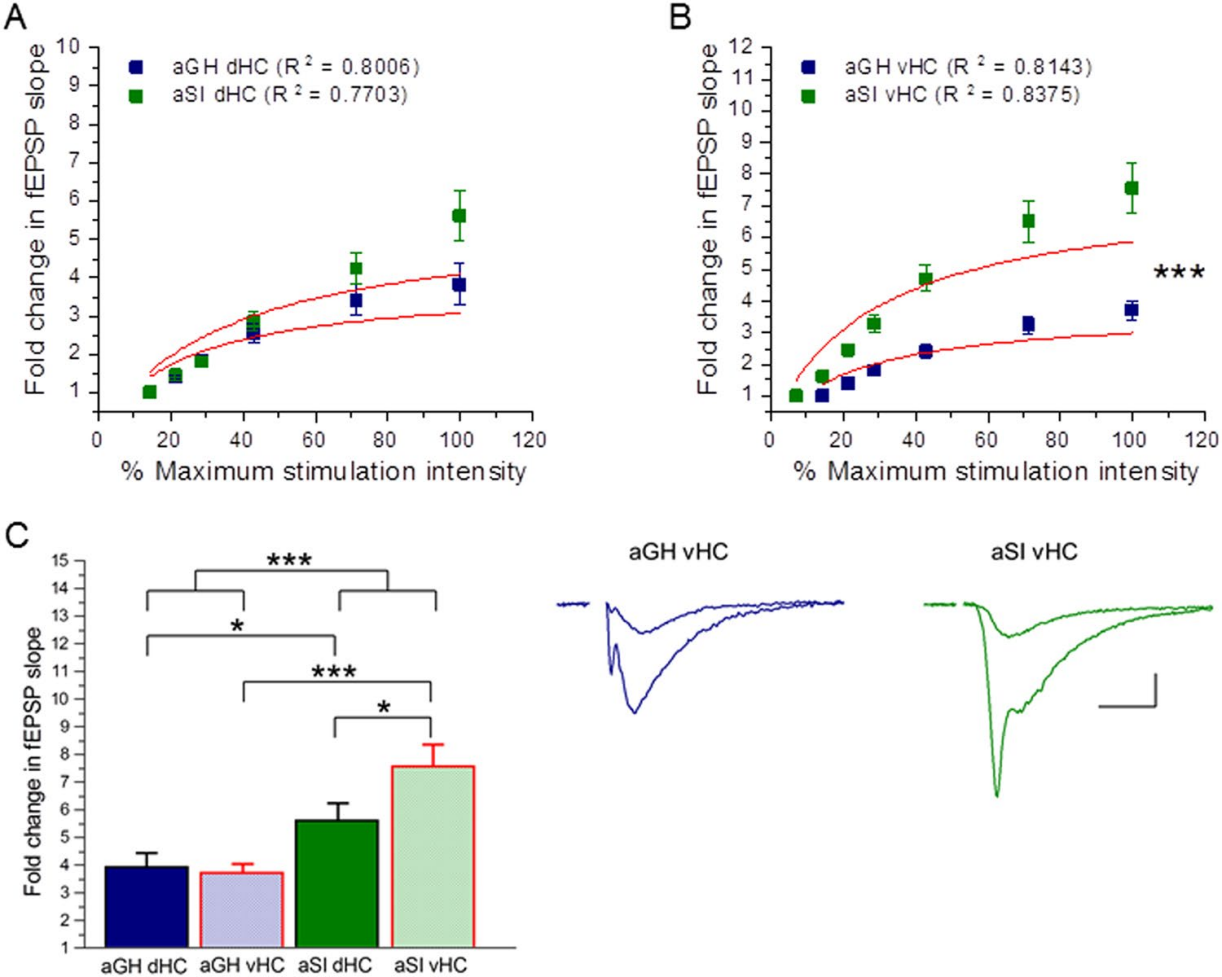
Adolescent social isolation has differential effects on baseline synaptic transmission along the hippocampal dorsoventral axis. (**A**) The input/output relationship is not different between dHC slices from aGH and aSI rats (aGH dHC: n = 27 slices, 16 rats; aSI dHC: 21 slices, 13 rats; F_(1, 10)_ = 0.91, p = 0.36), F-test. (**B**) vHC slices from aSI rats display a significant enhancement in the input/output function compared to aGH rats (aGH vHC: 33 slices, 13 rats; aSI vHC: 13 slices, 15 rats; F_(2, 10)_ = 18.37, p < 0.001, F-test). (**C**) Comparison of fold change in fEPSP slope at the maximum stimulation intensity shows that aSI enhances synaptic responses enhances synaptic responses in both the dHC and vHC, with the vHC showing a greater enhancement (effect of housing: p < 0.001, two-way RM ANOVA; aGH dHC vs. aGH vHC: p = 0.40; aSI dHC vs. aSI vHC: p = 0.02, aGH dHC vs. aSI dHC: p = 0.04, aGH vHC vs. aSI vHC: p < 0.001, Student-Neuman-Keuls *post hoc*). Representative traces display synaptic fEPSP responses at threshold and maximum stimulation intensities (Scale bars: 0.2 mV, 10 ms).Stimulus artifacts have been removed for clarity.﻿

The data analyses discussed in the previous sections were performed with dorsal and ventral hippocampal slices pooled together. With the functional segregation of the hippocampus along the dorsoventral axis in mind, we therefore performed additional analyses of our electrophysiological data, stratifying by hippocampal region. Figure 2 shows examples and coordindate ranges^48^ of slices taken from dorsal and ventral hippocampus used in our extracellular field recordings. In the dHC, slices from aGH and aSI rats displayed comparable input/output relationships (Fig. 3A; aGH dHC: n = 27 slices, 16 rats; aSI dHC: n = 21 slices, 13 rats). Further, comparisons of Michaelis-Menten fits to input/output curves were not different from each other (Fig. 3A; aGH dHC: R^2^ = 0.8006, aSI dHC: R^2^ = 0.7703, F_(1,10)_ = 0.91, p = 0.36, F-test). In marked contrast to the lack of an effect of adolescent social isolation on dHC transmission, in the vHC, slices from aSI rats showed significantly greater fold changes in fEPSP slope over threshold than slices from aGH rats (Fig. 3B; aGH vHC: n = 33 slices, 13 rats; aSI vHC: n = 23 slices, 15 rats). A comparison of Michaleis-Menten fits indicate that the input/output curves were different from each other (Fig. 3B; aGH vHC: R^2^ = 0.8143, aSI vHC: R^2^ = 0.8375, F_(2,10)_ = 18.37, p < 0.001, F-test). As shown in Fig. 3C, a comparison of fold change in fEPSP slope at the maximum stimulation intensity shows that adolescent social isolation enhances synaptic responses in both the dHC and vHC, with the vHC showing a greater enhancement (effect of housing: p < 0.001, two-way RM ANOVA; aGH dHC v aGH vHC: p = 0.40; aSI dHC v aSI vHC: p = 0.02, aGH dHC v aSI dHC: p = 0.04, aGH vHC v aSI vHC: p < 0.001, Student-Neuman-Keuls *post hoc*). Table 1 lists average fold changes in fEPSP slope at the maximum stimulus intensity for the groups compared in Fig. 3C. These data support the interpretation that adolescent social isolation primarily enhances baseline synaptic transmission in the ventral, but not dorsal, hippocampus.

**Table 1:** Fold change in fEPSP slope at maximum stimulus intensity.

	Mean	±SEM
aGH pooled	3.82	0.36
aSI pooled	6.60	0.42
aGH dHC	3.92	0.54
aGH vHC	3.72	0.49
aSI dHC	5.61	0.61
aSI vHC	7.58	0.59

**Figure 4:**
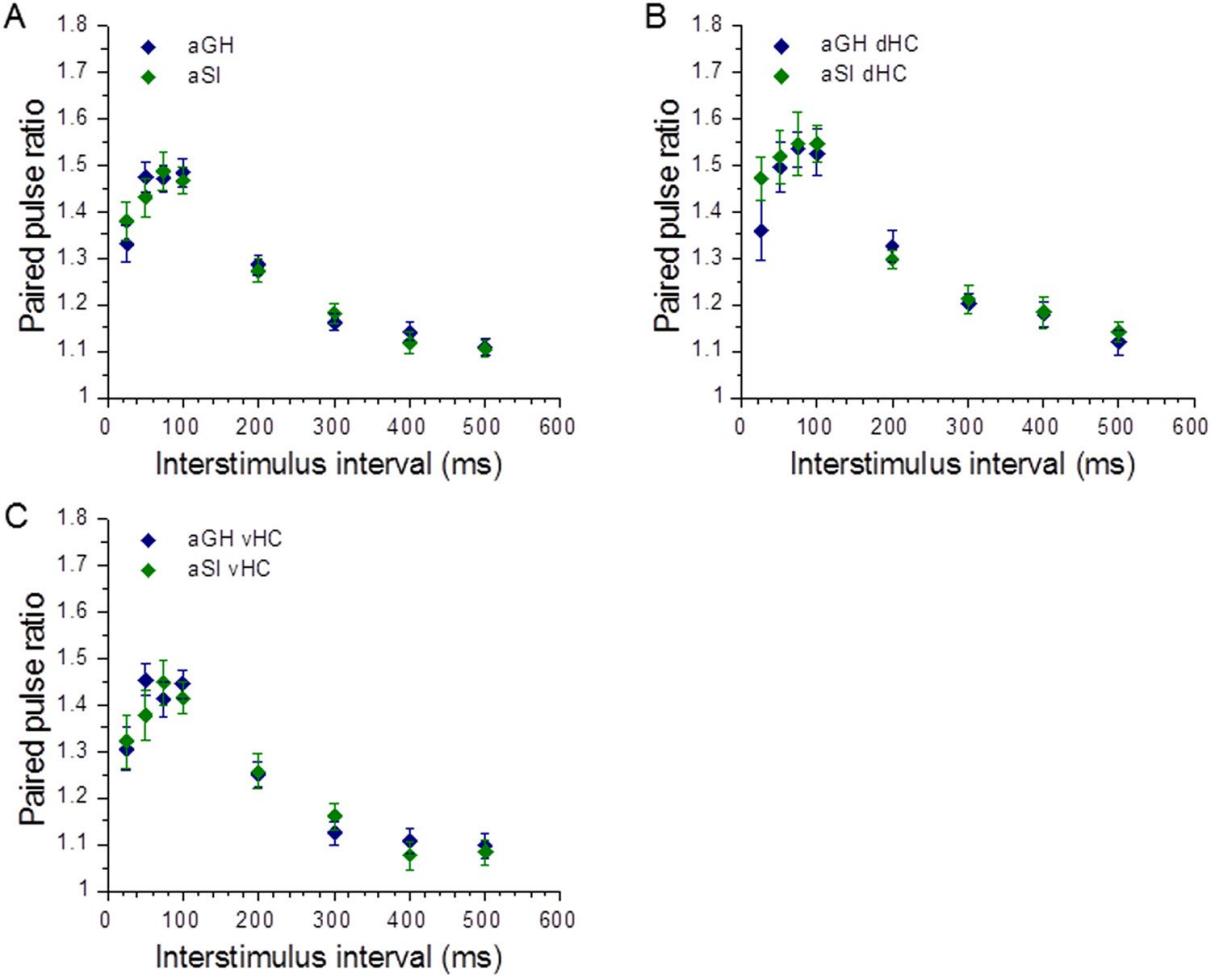
Adolescent social isolation does not affect short-term plasticity at hippocampal Schaffer collateral-CA1 synapses. (**A**) Analysis of pooled dorsal and ventral hippocampal slices does not show any differences in paired pulse ratios (aGH: n = 42 slices, 28 rats; aSI: n = 31 slices, 20 rats). **(B**) There are no differences in paired pulse ratios in dHC slices (aGH dHC: n = 20 slices, 14 rats; aSI dHC: n = 12 slices, 9 rats). (**C**) There are no differences in paired pulse ratios in vHC slices (aGH vHC: 22 slices,14 rats; aSI vHC: n = 19 slices, 11 rats).

Short-term plasticity reflects changes in synaptic strength occurring over time scales of milliseconds to minutes, and is generally thought to occur through presynaptic changes in neurotransmitter release^49^. Short-term plasticity can be observed by measuring responses to pairs of stimuli delivered at various interstimulus intervals. Hippocampal Schaffer collateral-CA1 synapses typically show short-term facilitation^50^ (i.e., paired pulse ratios >1). In paired pulse experiments from pooled dHC and vHC slices, we observed no differences in short-term plasticity (Fig. 4A; aGH: n = 42 slices, 28 rats; aSI: n = 31 slices, 20 rats). In the dHC, aGH and aSI slices displayed similar paired pulse ratios (Fig. 4B; aGH dHC: n = 20 slices, 14 rats; aSI dHC: n = 12 slices, 9 rats). In the vHC, slices from aGH and aSI rats again showed similar paired pulse ratios (Fig. 4C; aGH vHC: n = 22 slices, 14 rats; aSI vHC: n = 19 slices, 11 rats). These results suggest that adolescent social isolation does not affect presynaptic glutamate release at Schaffer-collateral-CA1 synapses in either the dorsal or ventral domains of the hippocampus.

### Reduced long-term potentiation arising from adolescent social isolation is largely confined to the ventral hippocampus

**Figure 5:**
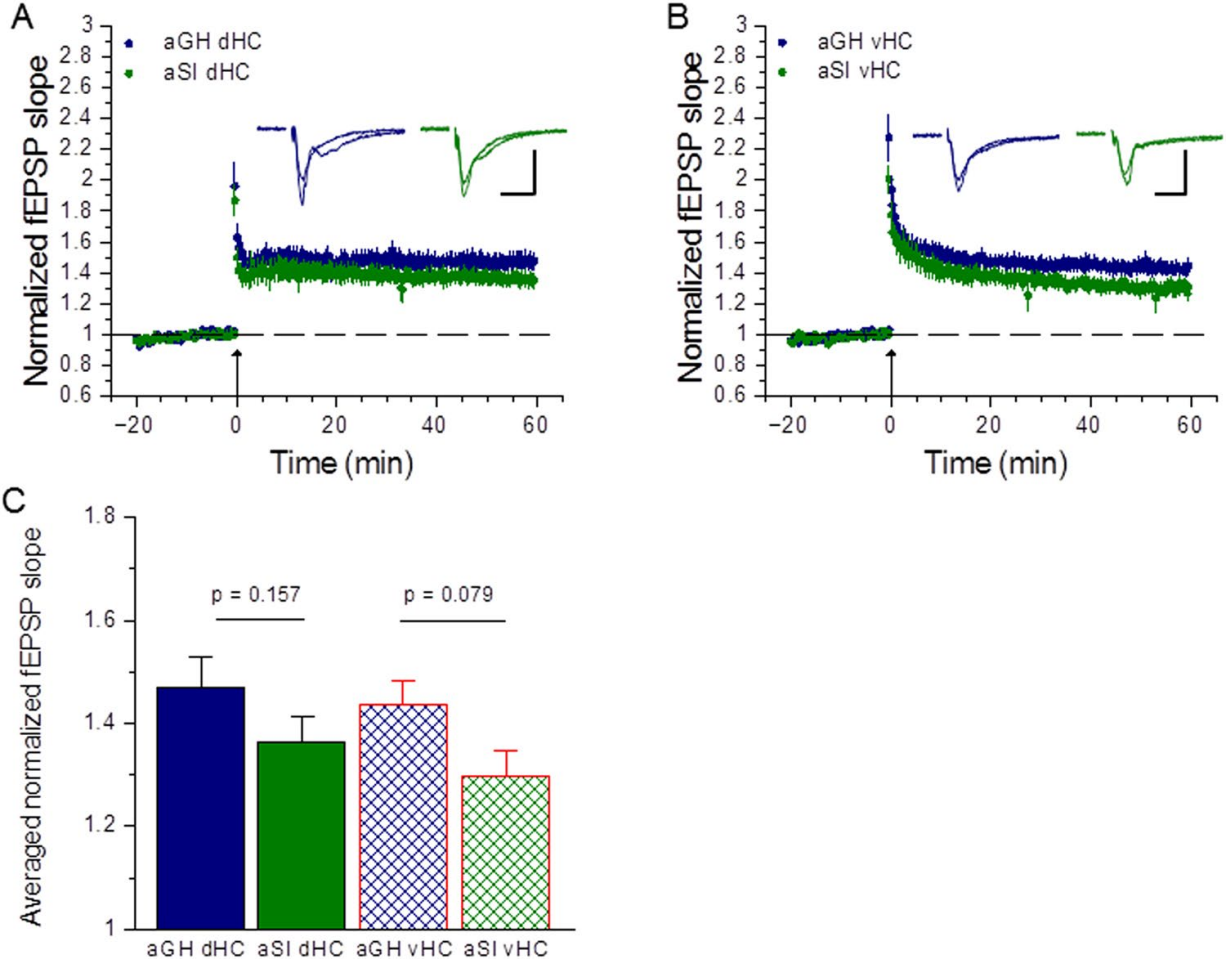
Adolescent social isolation reduces LTP in the ventral hippocampus. (**A**) TBS elicits comparable LTP magnitudes in dorsal hippocampal slices from aGH and aSI rats (aGH dHC: n = 23 slices, 15 rats; aSI dHC: 19 slices, 12 rats). (**B**) vHC slices from aSI rats display a strong trend toward lower LTP magnitudes than aGH rats (aGH vHC: 27 slices, 13 rats; aSI: n = 15 slices). In (**A**) and (**B**), arrow indicates delivery of TBS. Insets show representative traces before and after LTP induction. Stimulus artifacts have been removed for clarity (Scale bars: 0.5 mV, 20 ms). (**C**) Comparison of averaged normalized fEPSP slopes for the last ten minutes of recording (aGH dHC v aSI dHC: p = 0.157; aGH vHC v aSI vHC: p = 0.079, two-way RM ANOVA with Student-Neuman-Keuls *post hoc*).

We next extended our data analysis to evaluate differences in TBS-induced LTP levels by hippocampal regions. In dHC slices, TBS appeared to elicit lower levels of LTP in aSI rats than aGH rats, however, this difference was not statistically significant (Fig. 5A and C; aGH dHC: 1.47 ± 0.050, n = 23 slices, 15 rats; aSI dHC: 1.36 ± 0.055, n = 19 slices, 12 rats; p = 0.157, Student-Neuman-Keuls *post hoc* from two-way RM ANOVA). In the vHC, slices from aSI rats displayed a stronger trend toward lower LTP magnitudes that approached statistical significance (Fig. 5B and C; aGH vHC: 1.44 ± 0.047, n = 27 slices, 13 rats; aSI vHC: 1.30 ± 0.062, n = 15 slices, 7 rats; p = 0.079, Student-Neuman-Keuls *post hoc* from two-way RM ANOVA). As reported in Fig. 1C, a repeated measures two-way ANOVA revealed a main effect of housing (aGH vs. aSI: F_(1,80)_ = 5.17, p = 0.026), however, this analysis also revealed no effect of hippocampal region (dHC vs. vHC: F_(1,80)_ = 0.88, p = 0.351) nor an interaction between factors (housing X region: F_(1,80)_ = 0.084, p = 0.773). These observations suggest that adolescent social isolation results in reduced LTP that is preferentially localized within the vHC.

### Comparison of dorsal and ventral hippocampal baseline synaptic transmission and short-term plasticity within adolescent group housed and adolescent socially isolated housing conditions

**Figure 6:**
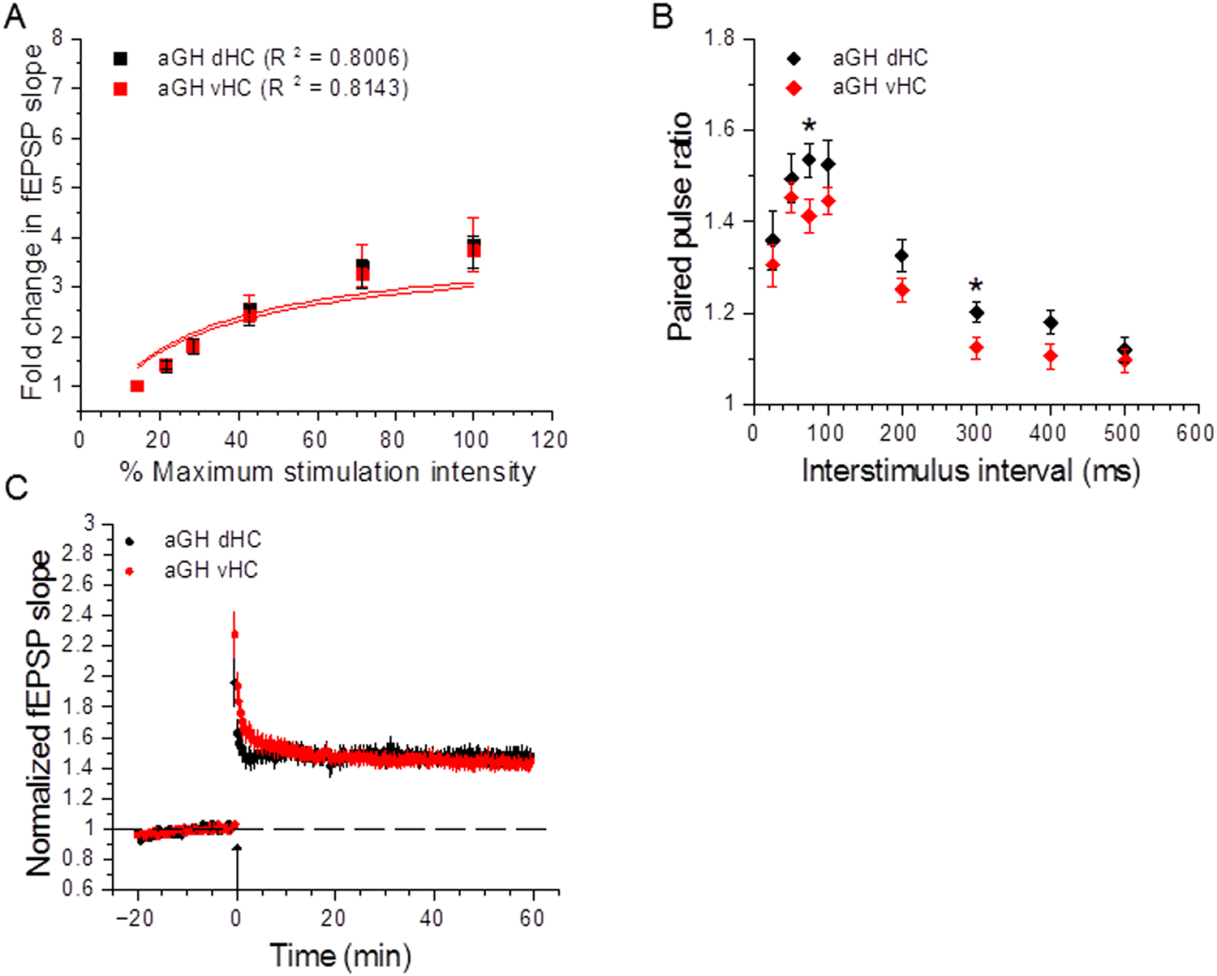
Comparison of dorsal and ventral hippocampal slices within adolescent grouped housed rats. (**A**) Dorsal and ventral hippocampal slices from aGH rats display similar input/output relationships (aGH dHC: n = 27 slices, 16 rats; aGH vHC: 20 slices, 14 rats; F_(1, 10)_ = 0.53, p = 0.48, F-test). (**B**) Short-term plasticity studies reveal lower paired pulse ratios at 75 ms and 300 ms interstimulus intervals in vHC slices compared to dHC slices in aGH rats (aGH dHC: 20 slices, 14 rats; aGH vHC: n = 22 slices, 14 rats; 75 ms: t(40) = 2.30, p = 0.027; 300 ms: t(40) = 2.37, p = 0.023). (**C**) There are no differences in TBS-induced LTP between dorsal and ventral hippocampal slices from aGH rats (aGH dHC: n = 23 slices, 15 rats; aGH vHC: n = 27 slices, 13 rats; p = 0.61, two-way RM ANOVA with Student-Neuman-Keuls *post hoc*). Arrow indicates delivery of TBS.

We extended our data analysis even further to compare dorsal and ventral hippocampal synaptic function and plasticity within each housing condition. In aGH rats, we found no differences in the input/output function between hippocampal regions (Fig. 6A; aGH dHC: n = 27 slices, 16 rats; aGH vHC: n = 33 slices, 13 rats; F_(1, 10)_ = 0.53, p = 0.48, F-test). Short-term plasticity experiments showed a trend toward lower paired pulse ratios in vHC slices (Fig. 6B; aGH dHC: n = 20 slices, 14 rats; aGH vHC: n = 22 slices, 14 rats), with statistically significant differences at the 75 ms (aGH dHC: 1.53 ± 0.037; aGH vHC: 1.41 ± 0.038; t(40) = 2.30, p = 0.027, Student’s t-test) and 300 ms (aGH dHC: 1.20 ± 0.023; aGH vHC: 1.12 ± 0.024; t(40) = 2.37, p = 0.023, Student’s t-test) interstimulus intervals. Together, these results suggest that under our adolescent group housing conditions, baseline synaptic transmission at Schaffer collateral-CA1 synapses are similar in both dorsal and ventral hippocampal regions, whereas there is a trend toward lower paired pulse facilitation in the vHC.

**Figure 7:**
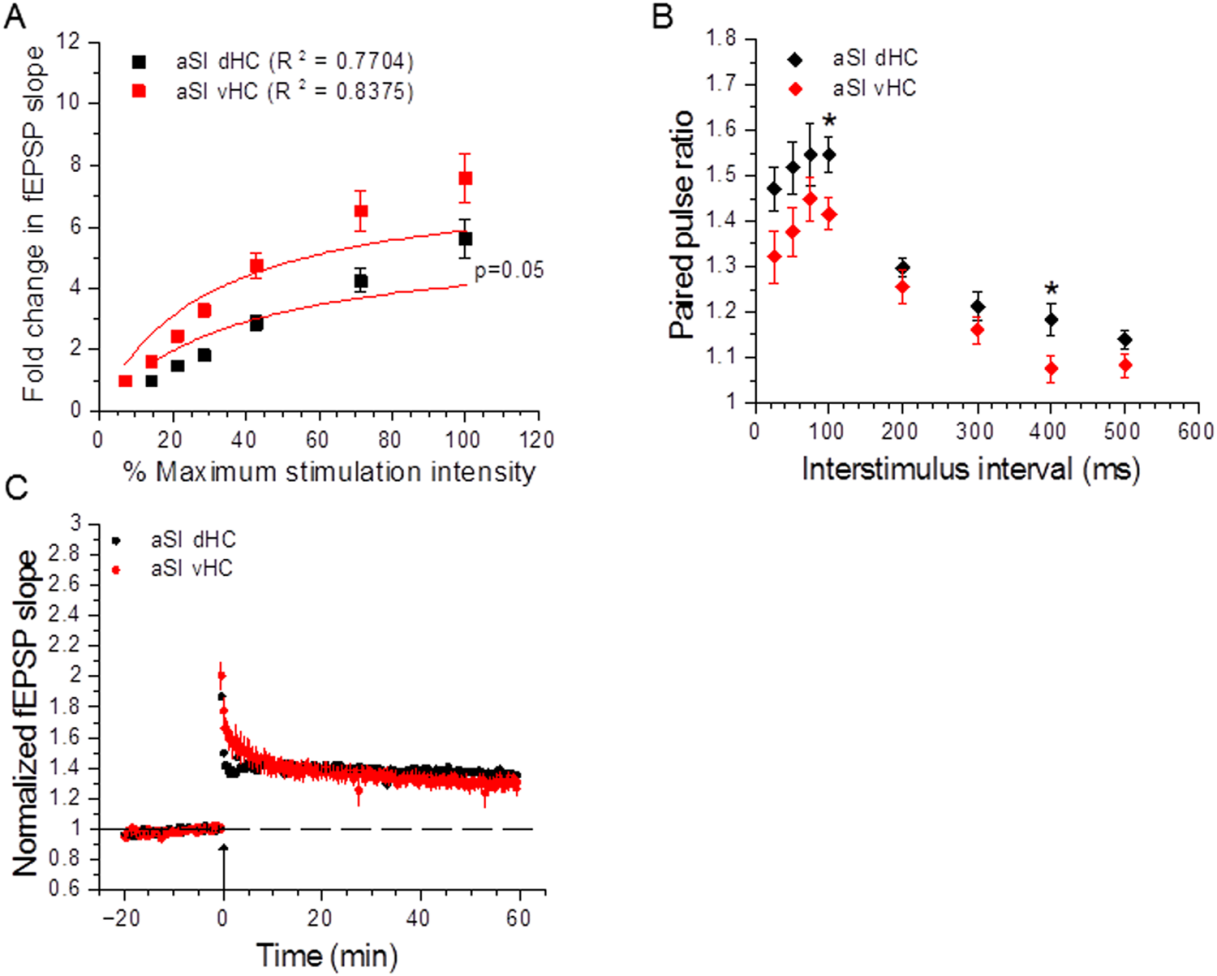
Comparison of dorsal and ventral hippocampal slices within adolescent socially isolated rats. (**A**) vHC slices from aSI rats display a strong trend toward enhanced input/output relationship compared to dHC slices (aSI dHC: n = 21 slices, 13 rats; aSI vHC: n = 23 slices, 15 rats; F_(1, 11)_ = 4.70, p = 0.05, F-test). (**B**) vHC slices from aSI rats show lower paired pulse ratios at 100 ms and 400 ms interstimulus intervals (100 ms: t(29) = 2.43, p = 0.021; 400 ms: t(29) = 2.33, p = 0.027). (**C**) There are no differences in TBS-induced LTP between dorsal and ventral hippocampal slices from aSI rats (aSI dHC: n = 19 slices, 12 rats; aSI vHC: 15 slices, 7 rats; p = 0.43, two-way RM ANOVA with Student-Neuman-Keuls *post hoc*). Arrow indicates delivery of TBS.

In aSI rats, we observed an increased input/output relationship in vHC slices compared to dHC slices that approached statistical significance (Fig. 7A, aSI dHC: n = 21 slices, 13 rats; aSI vHC: n = 23 slices, 15 rats; F_(1, 11)_ = 4.70, p = 0.05, F-test). Short-term plasticity experiments revealed a trend toward lower paired pulse ratios in vHC slices (Fig. 7B; aSI dHC: n = 12 slices, 9 rats; aSI vHC:n = 19 slices, 11 rats), with statistically significant differences at the 100 ms (aSI dHC: 1.55 ± 0.039; aSI vHC: 1.42 ± 0.035; t(29) = 2.43, p = 0.021, Student’s t-test) and 400 ms (aSI dHC: 1.18 ± 0.035; aSI vHC: 1.08 ± 0.029; t(29) = 2.33, p = 0.027, Student’s t-test) interstimulus intervals. These data suggest that adolescent social isolation enhances baseline synaptic transmission in the vHC. The observation that aSI vHC, like aGH ﻿vHC slices, also showed a trend toward lower paired pulse ratios than dHC under our adolescent socially isolated housing conditions further suggests that adolescent housing condition does not affect properties of presynaptic neurotransmitter release.

### Dorsal and ventral hippocampal Schaffer-collateral-CA1 synapses respond similarly to theta-burst stimulation

Several previous studies have reported marked differences in plasticity induced by tetanic stimulation between the dorsal and ventral domains of the hippocampus^51–59^. We therefore analyzed our data to compare TBS-induced LTP between dHC and vHC within each housing condition. In aGH rats, TBS elicited comparable levels of LTP in dHC and vHC slices (Fig. 6C; aGH dHC: 1.47 ± 0.050, n = 23 slices, 15 rats; aGH vHC: 1.44 ± 0.047, n = 27 slices, 13 rats; p = 0.612, Student-Neuman-Keuls *post hoc* from two-way RM ANOVA). Likewise, in aSI rats, we observed no differences in LTP magnitudes between dHC and vHC slices (Fig. 7C; aSI dHC: 1.36 ± 0.055, n = 19 slices, 12 rats; aSI vHC: 1.30 ± 0.062, n = 15 slices, 7 rats; p = 0.430, Student-Neuman-Keuls *post hoc* from two-way RM ANOVA). These data suggest that, at least under our recording conditions, dorsal and ventral hippocampal Schaffer-collateral-CA1 synapses do not differ in the induction or expression of TBS-induced LTP.

## Discussion

The most important findings from this study are that adolescent social isolation leads to a significant increase in synaptic excitability at hippocampal Schaffer collateral-CA1 synapses and that this effect is largely localized to the vHC. Extracellular field recordings revealed that adolescent social isolation results in increased baseline synaptic transmission in the vHC, and that this enhanced excitability is associated with a decrease in the magnitude of theta-burst-induced LTP. In contrast, dorsal hippocampal slices from both aGH and aSI rats displayed comparable baseline synaptic transmission, short-term plasticity, and levels of LTP. These results extend our characterization of neurobiological substrates mediating the behavioral and neurophysiological maladaptations arising from adolescent social isolation to include the vHC. In addition, these results provide additional neurophysiological evidence supporting the idea that the hippocampus is a set of functionally segregated domains. Moreover, the adaptive changes we observe in the vHC, regarded as the “emotional” region of the hippocampus, are consistent with burgeoning evidence that adolescent social isolation induces adaptations in brain regions that regulate affective states.

Our laboratory and others have commonly observed that rats socially isolated during adolescence exhibit a host of behavioral phenotypes that are consistent with increased risk of developing alcohol addiction and other mood disorders, like generalized anxiety disorder and post-traumatic stress disorder (PTSD)^21^; thus, we have posited the validity of the aSI model as a rodent model of heightened vulnerability to alcohol addiction and comorbid affective disorders^5^. Our initial effort at elucidating neurophysiological adaptations arising from adolescent social isolation has focused on the BLA, a brain region that is integral to the gating of stress, fear, and anxiety-like behaviors and also plays a role in motivation^60^ and alcohol self-administration^61,62^. For example, we have recently reported increased BLA pyramidal cell intrinsic excitability in aSI rats, and that local pharmacological inhibiton of the intrinsic excitability of these cells decreases anxiety-like behaviors^12^. Further, we have also revealed that adolescent social isolation is associated with deficits in amygdala-dependent extinction of learned fear^63,64^. As extensive anatomical studies have described robust reciprocal excitatory connectivity between the BLA and the vHC^25–30,39^, and mounting functional evidence is providing much insight into this circuit’s role in mediating emotionally-related behaviors^10,31–39^, it is likely that perturbations in the BLA-vHC circuit may contribute to the behavioral phenotypes engendered by the aSI model. The dearth of information on how amygdaloid-hippocampal circuitry is affected by adolescent social isolation and its potential role in alcohol addiction served as the impetus to begin an examination of this model’s effects on hippocampal synaptic transmission and synaptic plasticity.

A large body of literature demonstrates that exposure to stress elicits a multitude of adaptations in synaptic function and plasticity in the hippocampus^13,15,17,65^. Indeed, our extracellular field recordings demonstrate that chronic social isolation stress in adolescence result in lower LTP magnitudes at hippocampal Schaffer collateral-CA1 synapses. This observation is in agreement with results showing that socially isolated mice had reduced levels of hippocampal LTP^66^. In addition, the reduced LTP magnitudes we observe arising from chronic social isolation stress are congruent with hippocampal LTP impairments observed in rats exposed to acute stress paradigms^13,15,17^. Whereas Sanna *et al*.^66^ observed decreased baseline synaptic transmission as measured by input/output curves in adolescent socially isolated mice, we found a significant enhancement of the input/output function in our aSI rats. This observation is in line with previous reports that exposure to chronic stressors leads to enhanced excitatory responses in the hippocampus^14^ and in the BLA^11,12^. It is possible that differences in hippocampal electrophysiological properties between rats and mice can account for these observations^67^. Short-term plasticity studies revealed comparable paired pulse facilitation between aGH and aSI rats. Altogether, these data suggest that neuroadaptations arising from adolescent social isoloation may impinge upon mechanisms underlying excitatory synaptic transmission and synaptic plasticity, and further point towards a postsynaptic locus for these adaptations.

It is becoming increasingly appreciated that the hippocampus is not a monolithic brain structure supporting cognition, but rather, can be thought of as a set of functionally separate domains, with the dorsal region mediating declarative memories and the ventral region mediating certain types of emotional memories^7,22–24^. With this dichotomy in mind, we stratified the analysis of our electrophysiological data to examine the effects of housing condition within each hippocampal subregion. A comparison of dHC slices from aGH and aSI rats showed that properties of baseline synaptic transmission did not differ between housing conditions; in addition, short term plasticity was not affected in this hippocampal subregion. Further, adolescent social isolation did not affect LTP induction and expression, as dHC slices from aGH and aSI rats exhibited similar levels of potentiation. Analysis of vHC slices from aGH and aSI rats, on the other hand, revealed two prominent effects of chronic social isolation during adolescence. First, input/output measurements in vHC slices showed markedly increased baseline synaptic transmission in aSI rats compared to aGH rats. Specifically, we found that adolescent social isolation resulted in larger fold changes in fEPSP slopes over the threshold stimulus intensity producing a synaptic response. Second, we observed a strong trend toward smaller LTP magnitudes in vHC slices from aSI rats relative to aGH rats. Although the difference in LTP magnitudes in the vHC was not found to be statistically significant, most likely due to biological variability arising from the activation of multiple circuits within the vHC^7,25–28,30,39,68^ in our electrophysiological recording conditions, these data support the interpretation that chronic social isolation stress in adolescent rats leads to an enhancement in baseline excitatory synaptic transmission, and that this enhanced excitability may occlude the induction and expression of LTP at vHC Schaffer collateral-CA1 synapses. Similar to our findings in the dHC, short-term plasticity was also not affected in vHC slices from aGH and aSI rats. These observations further suggest that adolescent social isolation does not affect presynaptic glutamate release at Schaffer-collateral-CA1 synapses in either the dorsal or ventral domains of the hippocampus.

While a number of pioneering behavioral studies using targeted dHC and vHC lesions and pharmacological inactivation have provided great insight into elucidating the functional organization of the hippocampus along the dorsoventral axis^7,23,24^, fewer studies have investigated electrophysiological differences between the dHC and vHC. We therefore further analyzed our data to assess dorsoventral differences within each housing condition. Within aGH rats, a comparison of baseline synaptic transmission in dHC and vHC slices showed no differences in the input/output function. In aSI rats, however, we observed enhanced baseline synaptic transmission in vHC slices. Within both aGH and aSI groups, our evaluation of short-term plasticity revealed an overall trend toward lower paired pulse ratios in vHC slices compared to dHC slices, with statistically significant differences at several interstimulus intervals. In the extended analysis of our LTP experiments, we found no dorsoventral differences in LTP magnitudes within either housing condition. Collectively, these observations strengthen the interpretation that the effects of adolescent social isolation on hippocampal synaptic function and synaptic plasticity have a postsynaptic locus and are restricted to the vHC.

In relation to previous studies that have described distinct dorsoventral differences in synaptic function and synaptic plasticity^51–59^, our analysis revealed a number of similarities and apparent differences. First, our observations of no dorsoventral differences in the input/output function agree with results obtained by other groups in both rats and mice^55–59^. Second, while both *in vivo* ^54^ and *in vitro* ^51–53^ recordings report demonstrably smaller levels of short-term facilitation in the vHC, we only observed a trend toward lower paired pulse rations in the vHC, although with statistical significance at a few interstimulus intervals. Third, a consistent observation from several laboratories^51–59^ is that the vHC exhibits lower LTP magnitudes than the dHC. Our observations that LTP magnitudes were not different between hippocampal regions are at odds with these prior reports.

A comparison of the effects of stress on synaptic function and synaptic plasticity along the dorsoventral axis of the hippocampus has also yielded conflicting results. Maggio and Segal have reported differential effects of a variety of acute stressors (forced swim, inescapable platform, and restraint stress) on hippocampal function^56,58^. First, they report no dorsoventral effects of these acute stressors on baseline synaptic transmission. In contrast, in our chronic adolescent social isolation stress model, we found a striking enhancement of baseline synaptic transmission in the vHC. Next, they have also reported diametrically opposite effects of acute stress on LTP, with the dHC displaying decreased LTP magnitudes, and the vHC expressing increased LTP magnitudes. On the other hand, we found that the aSI model produced a reduction of LTP magnitudes that was most prominent in the vHC. Our results do confirm, however, other reports that both acute and chronic stress impairs the induction of LTP in the hippocampus^13,15,17,66,69^. The disparity of these observations highlight the necessity to gain a better understanding of the underlying mechanisms that may be engaged by various stressors along the dorosoventral axis of the hippocampus.

In addition to the aforementioned species and stress model differences, other methodological differences between these prior studies and our present work may have also contributed to these discordant observations. It is important to note that precise anatomical and physiological definitions of dorsal versus ventral hippocampal domains are lacking, and it has been recently proposed that the hippocampus may be more appropriately conceptualized as a continuum of functional gradients along the dorsoventral axis, rather than discrete domains^24^. The studies from the Kostopoulos^51–53^ and Segal^55–59^ laboratories, for example, only used slices restricted to ~3 mm of the dorsal and ventral poles of the hippocampus, whereas we used slices encompassing the entire hippocampal formation in our experiments. As such, slices derived from “intermediate” hippocampus, which seems to display characteristics overlapping those of dHC and vHC^7,24^, were likely included in both dorsal and ventral analyses. Future work rigorously investigating the electrophysiological properties of this “intermediate” domain of the hippocampus is certainly needed.

The LTP induction protocols used in the long-term plasticity experiments discussed here present another major methodological difference. All of the previously published LTP experiments discussed thus far utilized high-frequency stimulation (HFS; e.g., 100 Hz train delivered for 1 s) to elicit LTP^51–59^. While HFS patterns are regarded as standard LTP induction protocols, these protocols have been argued to be supraphysiological^40,41^. The theta-burst stimulation (TBS) protocol we used in our studies mimics the complex-spike discharges and modulation of pyramidal cell excitability obtained from *in vivo* recordings from awake, behaving animals performing learning tasks. Thus, TBS is widely considered to be a physiologically relevant stimulus protocol^38,39^. Moreover, mounting lines of evidence demonstrate that hippocampal theta rhythm is critical to communication in circuits linking the vHC to the BLA and the medial prefrontal cortex (mPFC) in the encoding of fear memories and the gating of anxiety-like behaviors and social interaction behaviors^43–45^.

Another important consideration is the behavioral state of the control animals prior to performing experiments. While the studies discussed above^51–59^ and the studies we report here all used commercially-sourced animals as controls, our control rats were group housed for at least six weeks before recordings commenced. As we have previously shown that commercially-sourced rats present with phenotypes closely resembling aSI rats^70^, it is possible that our group housing protocol itself induces some form of plasticity. Our ongoing characterization of the aSI model will provide further insight into compensatory mechanisms invoked in both group housed and socially isolated housing conditions.

We have previously reported that adolescent social isolation leads to a significant increase in the intrinsic excitability of BLA pyramidal neurons^12^. Underlying this enhanced excitability is altered expression and function of small-conductance Ca2+-activated K+ (SK) channels arising from adolescent social isolation. Specifically, BLA SK channel subunit expression is reduced in aSI rats compared to aGH rats, which is further associated with increases in BLA pyramidal cell excitability^12^. We further demonstrated that positive modulation of SK channel activity can reduce anxiety-like behavior^12^. Importantly, this is the one neural adaptation that we have shown to be similar in adolescent socially isolated and adult commercially sourced rats^12^. While the role of SK channels in mediating intrinsic excitability is well-established, SK channels have garnered much recent attention in the synaptic function, cognition, and addiction fields^71^. In the hippocampus, a host of studies has demonstrated that blocking SK channel activity can decrease TBS-LTP thresholds, increase TBS-LTP ceilings, and enhance performance in hippocampus-dependent behavioral tasks; conversely, increasing SK channel activity and/or expression is associated with impaired LTP induction and performance in behavior tasks^72–81^. Moreover, differences in SK channel expression and function underlying LTP induction between the dorsal and ventral domains of the hippocampus have recently been reported^82^. Together, these observations implicate SK channels as a molecular target that may contribute to the synaptic adaptations arising from adolescent social isolation.

Altogether, our results extend our characterization of neurobiological substrates perturbed by chronic adolescent social isolation stress to include the vHC. Our observations of enhanced excitation and impaired synaptic plasticity are consistent with and parallel to our findings from ongoing studies in the BLA. These data support an important role for the BLA-vHC circuit in the generation of behavioral phenotypes observed in the aSI model, which would help to further strengthen the validity of the aSI model as a model of addiction vulnerability. The use of chemogenetic and/or optogenetic approaches will provide invaluable insight into the precise roles of the BLA-vHC circuit in the maladaptive behaviors promoted by adolescent social isolation. Further, using chemogenetic/optogenetic techniques to interrogate specific ventral hippocampus-containing circuits may also help to resolve some of the disparities in the literature regarding how early life stress dyregulates synaptic communication in this brain region. Lastly, our results contribute additional lines of evidence to a growing body of literature demonstrating that the hippocampus exhibits a functional heterogeneity along its dorsoventral axis.

## Methods

### Adolescent social isolation

Male Long–Evans rats sourced from a commercial supplier (Envigo, Indianapolis, IN) arrived at postnatal day 21 (PD 21) and were group housed (4 rats/cage) in large Plexiglas cages (33.0 × 59.7 cm; Nalgene) for one week (until PD 28). Following this acclimation period, rats were assigned to either adolescent group housed (aGH) or adolescent socially isolated (aSI) housing conditions as previously described^12,63^. Rats assigned to the aGH group continued to be housed in groups of four until they were sacrificed for *ex vivo* electrophysiology experiments (PD 85–PD 109). Rats assigned to the aSI group were individually housed in smaller Plexiglas cages (25.4 × 45.7 cm) for at least six weeks (~PD 70) before performing electrophysiological experiments. aSI animals were exposed to the same olfactory, visual, and auditory cues as aGH rats but were deprived of social contact with peer rats during this period. Animals in both housing conditions were weighed and handled once per week. All animals had *ad libitum* access to food and water throughout the study. Animals were maintained on the same light/dark cycle and ate the same diet. All experiments were performed in accordance with the National Institutes of Health Guide for the Care and Use of Laboratory Animals, and all experimental protocols were approved by the Wake Forest University School of Medicine Animal Care and Use Committee.

### Electrophysiology

Brains from aGH and aSI rats were rapidly removed and 400 μm-thick coronal slices containing the hippocampus were prepared on a vibratome (Leica VT1000S, Leica Microsystems, Buffalo Grove, IL) while immersed in an ice-cold cutting solution containing (in mM): 85 NaCl, 3 KCl, 1.25 NaH_2_PO_4_, 25 NaHCO_3_, 0.5 CaCl_2_, 7 MgCl_2_, 10 D-glucose, 75 sucrose, and 0.6 ascorbate; pH was adjusted to 7.4, osmolarity to ~300 mOsm, and saturated with 95% O_2_/5% CO_2_. Slices were maintained for at least one hour submerged in an incubation chamber at room temperature (21–23 °C) before commencing electrophysiology experiments in artificial cerebrospinal fluid (ACSF) containing (in mM): 125 NaCl, 2.5 KCl, 1.25 NaH_2_PO_4_, 25 NaHCO_3_, 2 CaCl_2_, 1 MgCl_2_, 10 D-glucose; pH was adjusted to 7.4, osmolarity to ~300 mOsm, and saturated with 95% O_2_/5% CO_2_. Derivation of slices from dorsal (dHC) or ventral (vHC) hippocampus was noted prior to recording using operational definitions suggested by Maruki *et al*.^54^, and Fanselow and Dong^7^.

Extracellular field recordings were performed as previously described^83^. Slices were placed in a submersion chamber on an upright microscope (Olympus BX51WI, Olympus Scientific Solutions Americas, Center Valley, PA) heated to 30–32 °C, and were superfused with oxygenated ACSF at a flow rate of 2 mL/min. Borosilicate filamented glass recording electrodes (1–3 MΩ resistance) prepared from a vertical pipette puller (Narishige PC-10, Narishige International USA) and filled with ACSF were placed in *stratum radiatum* of area CA1, and stimuli were delivered (ISO-Flex, A.M.P.I., Jerusalem, Israel) via a nickel dichromate bipolar electrode positioned along the Schaffer collateral afferents from area CA3. Recordings were acquired with either an Axopatch 200B or Axoclamp 2B amplifier and digitized with a Digidata 1440 A or Digidata 1322 A (Molecular Devices, Sunnyvale, CA). Data were acquired, stored, and analyzed using pClamp 10.3 (Molecular Devices).

Input/output curves were generated by delivering test stimuli at 0.1 Hz and recording responses to increasing stimulus intensities (10–700 μA). Short-term plasticity was assessed by delivering pairs of stimulus pulses at interstimulus intervals ranging from 25–500 ms. For LTP experiments, stimulus intensity was set to 40–50% of the threshold for observing population spikes at the *stratum radiatum* recording electrode as determined by input/output curves. A minimum of 30 min of baseline stimulation (0.05 Hz) was recorded before LTP induction. LTP was induced by a theta-burst stimulation (TBS) protocol composed of trains of 10 stimulus bursts delivered at 5 Hz, with each burst consisting of four pulses at 100 Hz, delivered three times with a 20 s interstimulus interval^84^.

### Data Analysis

All recordings and initial analyses were performed blind to housing condition. A one millisecond window was used to measure the rise slopes of the downward-deflecting field excitatory postsynaptic potential waveform (i.e., fEPSP slopes). Input/output data were analyzed by normalizing fEPSP slopes at the threshold stimulation intensity for eliciting a synaptic response. Input/output plots were fit to a Michaelis-Menten model, with fits constrained to the highest value for each dataset; curve fits were compared by F-test. Paired pulse ratios were calculated by dividing the fEPSP slope of the response to the second pulse by the fEPSP slope of the response to the first pulse. LTP magnitudes were measured by averaging fEPSP slopes for the last ten minutes of the one hour post-TBS recording period. Data were analyzed using unpaired Student’s t-test and repeated measures two-way ANOVA (two-way RM ANOVA) with Student-Neuman-Keuls *post hoc* tests for comparison of means across housing conditions (aGH vs. aSI) and hippocampus regions (dorsal vs. ventral) where appropriate. All data are presented as mean ± SEM. The alpha level for all analyses was set at p < 0.05 (*p < 0.05, ***p < 0.001).
